# Alterations in Body Weight and Composition in Patients With Graves’ Disease Treated with Total Thyroidectomy

**DOI:** 10.7759/cureus.63338

**Published:** 2024-06-27

**Authors:** Takashi Fukuda, Seigo Tachibana, Tomohiro Osako, Yusuke Mori, Hisakazu Shindo, Hiroshi Takahashi, Shinya Satoh, Yuji Nagayama, Hiroyuki Yamashita

**Affiliations:** 1 Department of Endocrinology, Yamashita Thyroid Hospital, Fukuoka City, JPN; 2 Department of Endocrinology, Tomo Thyroid Clinic, Kagoshima City, JPN; 3 Department of Surgery, Yamashita Thyroid Hospital, Fukuoka City, JPN

**Keywords:** surgery, thyroidology, inbody, thyroidectomy, graves’ disease, body composition, body weight, thyroid

## Abstract

Objective: Thyrotoxicosis causes excess energy expenditure, resulting in weight loss, despite increased appetite, and changes in body composition, which are typically reversible with the normalization of thyroid hormone levels. However, patients with hyperthyroidism due to Graves’ disease are sometimes hesitant to undergo treatment because of the perceived morbidity associated with weight gain. Therefore, obtaining data to explain the details of such weight gain to these patients is important. This study aimed to investigate changes in body weight and composition in patients with Graves’ disease after total thyroidectomy.

Methods: In total, 21 patients with Graves’ disease who underwent total thyroidectomy were enrolled. Among them, nine patients were hyperthyroid (group A) and 12 were euthyroid (group B, control) immediately before surgery. Body weight, height, and body composition using bioelectrical impedance were measured preoperatively and five months postoperatively.

Results: In all patients, body weight, body mass index, and skeletal muscle mass, but not fat mass, significantly increased postoperatively. In individual groups, a significant increase in skeletal muscle and fat masses was observed solely in groups A and B, respectively. Furthermore, a significant positive correlation between preoperative thyroid function and differences in skeletal muscle mass preoperatively and postoperatively was found.

Conclusion: Our study shows that the normalization of thyroid function using thyroidectomy in patients with Graves’ disease is accompanied by weight gain, mainly due to an increase in skeletal muscle mass. These data are clinically significant because they enable physicians to explain to patients that weight gain after surgical treatment for Graves’ disease is favorable and reassure them of their concern.

## Introduction

Thyrotoxicosis causes excess energy expenditure, resulting in weight loss despite increased appetite and changes in body composition [1]. Body weight reduction caused by excess thyroid hormones is attributed to accelerated systemic catabolism, increased energy production, and higher oxygen consumption [2]. A short-term administration of triiodothyronine (T3) for two weeks to healthy patients decreased skeletal muscle mass [3], and a comparison of lean and fat masses between patients with hyperthyroidism due to Graves’ disease and euthyroid controls revealed a significant decrease in lean mass in patients with hyperthyroidism due to Graves’ disease [4].

Although these changes are generally reversible with the normalization of thyroid hormone levels, weight gain after treatment for Graves’ disease can sometimes lead to patients avoiding treatment due to concerns that the weight gain associated with treatment is unfavorable to their cosmetics and health. Therefore, obtaining data to explain the details of such weight gain to these patients is important from a clinical perspective. However, the results of studies examining alterations in body composition following treatment for Graves’ disease have been inconsistent. Several previous studies have compared changes in body composition in patients with hyperthyroidism due to Graves’ disease or toxic multinodular goiter pre- and post-treatment with antithyroid drugs (ATD) or radioactive iodine, showing increases in lean mass [5], fat mass [6], or both [1,2,7-9]. Furthermore, these changes in body composition have been reported to occur sequentially (muscle gain followed by fat gain or vice versa) [2,9] or concurrently [10]. However, only anecdotal reports of changes in body weight after surgical treatment for Graves’ disease in the early 2000s exist [11,12], and only one study reports alterations in body composition in three patients with Graves’ disease treated with ATD and surgery [9]. Therefore, this study aimed to investigate the effect of total thyroidectomy on body weight and composition in patients with Graves’ disease. 

## Materials and methods

This prospective study was conducted at Yamashita Thyroid Hospital, Fukuoka, Japan. The study was approved by the Ethics Committee of Yamashita Thyroid Hospital (approval number: 2023-2) and was conducted in accordance with the Declaration of Helsinki. Informed consent was obtained from all participants.

Patients

Overall, 21 of the 27 patients with Graves’ disease who underwent total thyroidectomy between June and August 2022 were enrolled in this study, and their body weight and body composition were assessed. Five patients were excluded because they were transferred before postoperative anthropometry and one was excluded because they underwent ablation therapy shortly after surgery. These patients underwent surgery due to adverse reactions to ATD medication, coexistence of cancer, giant goiter, desire for pregnancy, and difficulty in remission. Thyroxine (T4) replacement at body weight (kg) × 1.6 μg was started on the day after surgery. The diagnosis of hyperthyroidism was based on clinical assessment and biochemical findings of (free (f)T4 and fT3) above the reference range, thyroid-stimulating hormone (TSH) below the reference range, and anti-TSH receptor autoantibodies (TRAb)).

Measurements

Body Composition

Height and weight were measured preoperatively and five months postoperatively, and BMI (weight (kg)/height (m)^2^) was calculated. Body composition, including skeletal muscle and fat masses, was measured using bioelectrical impedance with an InBody 770 body composition analyzer (InBody Co., Ltd, South Korea) [2,10,13].

Laboratory Tests

Serum levels of fT4, fT3, TSH, and TRAb were measured using commercially available ECLusys kits (F. Hoffmann-La Roche AG, Basel, Switzerland). 

Statistical analysis

Data are expressed as mean±standard error (SE) and analyzed with Wilcoxon rank sum test using JMP software (version 17.2.0; JMP Statistical Discovery LLC, Cary, North Carolina, United States). Significant differences were considered at p<0.05.

## Results

Twenty-one patients with Graves’ disease were categorized into groups A (comprising nine patients who were hyperthyroid (fT3 ≥4.3 pg/mL) despite treatment with ATD, potassium iodide, and/or steroid) and B (including the remaining 12 patients who were euthyroid, immediately before surgery). Group B was considered the non-surgical control group since thyroid function was normalized using medical treatment preoperatively in the group. Therefore, the effect of surgical treatment alone should be observed in group A. The data for the entire cohort and each group are shown in Table 1. Preoperative TSH was significantly lower, and fT3 and fT4 were significantly higher in Group A than in Group B. All patients were euthyroid five months postoperatively (data not shown).

**Table 1:** Preoperative patient characteristics * reference range: 0.61–4.23; ** reference range: 0.9–1.7; *** reference range: 2.3–4.3 p-values in bold indicate statistical significance T: thiamazole; KI: potassium iodide; PTU: propylthiouracil; S: steroid; TSH: thyroid-stimulating hormone; fT4: free thyroxine; fT3: free triiodothyronine

	Total cohort (N=21)	Hyperthyroid group (Group A) (N=9)	Euthyroid group (Group B) (N=12)	P value (Group A vs. Group B)
Sex, male/female, n	6/15	2/7	4/8	0.66
Age (years), mean±SE	46±3.2 (20–78)	40.8±3.27 (20–78)	52±5.5 (26–57)	0.07
Preoperative TSH (μIU/mL)*, mean±SE	0.57±0.23	0.0±0.0	1.33±0.43	0.0002
Preoperative fT_4_ (ng/dL)**, mean±SE	1.46±0.17	1.85±0.25	0.93±0.057	0.0055
Preoperative fT_3_ (pg/mL)***, mean±SE	5.57±0.67	7.62±0.73	2.84±0.12	0.0001
Preoperative treatment (T:KI:T+KI:PTU+KI:T+KI+S:KI+S)	5:6:5:2:1:2	1:4:3:1:1:2	4:2:2:1:0:0	0.51
Preoperative treatment periods (months), mean±SE	59.7±15.3	52.8±19.9	69±24.7	0.062

Data for each measurement preoperatively and five months postoperatively are summarized in Table 2. In the entire cohort, all parameters, including body weight, BMI, and skeletal muscle mass, except for fat mass, increased significantly postoperatively. When analyzed between the two groups, although body weight and BMI increased significantly in both groups, only the increase in skeletal muscle mass in Group A and that in fat mass in Group B were significant. The differences in these parameters preoperatively and postoperatively were also calculated in each group and compared (Table 3). Only the difference in skeletal muscle mass gain between the two groups was significant.

**Table 2:** Comparison of changes in body weight and composition preoperatively and five months postoperatively. p-values in bold indicate statistical significance

	Total cohort			Hyperthyroid group (Group A)			Euthyroid group (Group B)		
	Pre-surgery, mean ± SE	Post-surgery, mean ± SE	P value	Pre-surgery, mean ± SE	Post-surgery, mean ± SE	P value	Pre-surgery, mean ± SE	Post-surgery, mean ± SE	P value
Body weight (kg)	55.7±2.4	58.4±2.3	0.0003	54.5±3.2	58.1±3.2	0.039	57.3±3.7	58.9±3.6	0.039
BMI (kg/m^2^)	21.7±0.67	22.8±0.65	0.0003	20.8±0.62	22.2±0.62	0.039	22.8±1.3	23.5±1.3	0.039
Skeletal muscle (kg)	21.3±1.2	22.6±1.	0.0013	21.3±1.8	23.3±1.9	0.0063	21.4±1.5	21.7±1.5	0.073
Fat mass (kg)	16.1±1.2	17.0±0.96	0.0542	14.9±0.9	15.5±0.8	0.3877	17.7±1.8	18.9±1.9	0.018

**Table 3:** Comparision of preoperative and postoperative differences in body composition p-values in bold indicate statistical significance SE: standard error

Characteristics	Total cohort, mean ± SE	Hyperthyroid group (Group A), mean ± SE	Euthyroid group (Group B), mean ± SE	P value (Group A vs. Group B)
Difference in body weight (kg)	2.8±0.63	3.6±0.95	1.6±0.61	0.12
Difference in BMI (kg/m^2^)	1.1±0.25	1.4±0.37	0.67±0.27	0.15
Difference in skeletal muscle mass (kg)	1.3±0.35	2.0±0.48	0.3±0.26	0.012
Difference in fat mass (kg)	0.88±0.43	0.68±0.66	1.1±0.50	0.86

**Figure 1:**
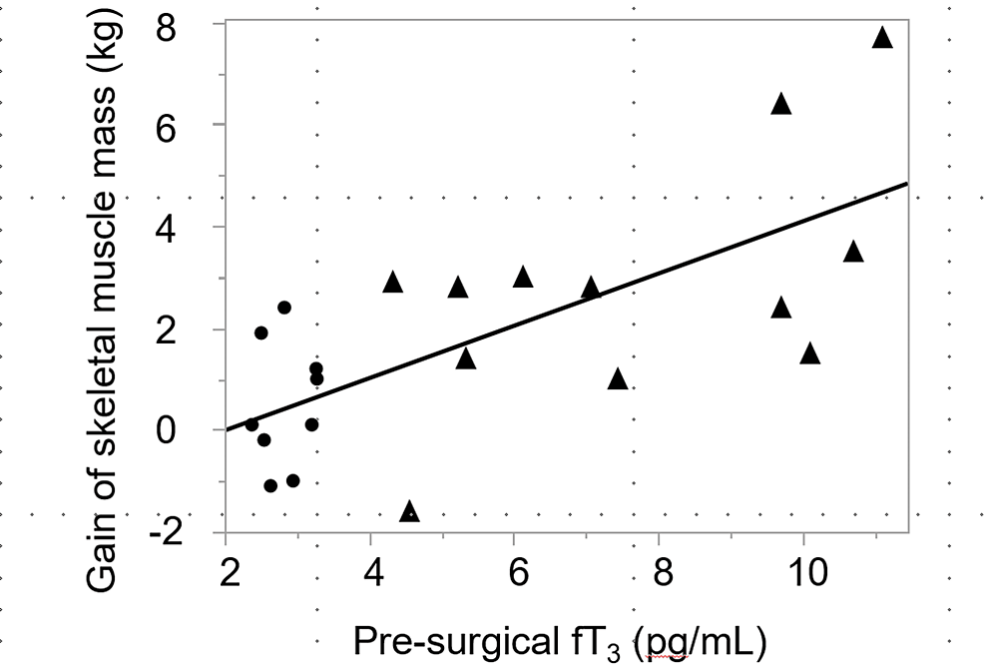
Correlation between pre-surgical thyroid function (fT3) and gain of skeletal muscle mass after surgery. Black circles indicate hyperthyroid patients and triangles indicate euthyroid patients before surgery fT3: free triiodothyronine

Correlations between preoperative thyroid function (fT3) and differences in skeletal muscle mass preoperatively and postoperatively were calculated. As shown in Figure 1, significant positive correlations were observed between changes in skeletal muscle mass and preoperative fT3 levels (y=-0.831+0.381x, r=0.737, p=0.0001).

## Discussion

To the best of our knowledge, this is the first study to report that surgical therapy for patients with Graves’ disease as a whole significantly increased body weight, BMI, and skeletal muscle mass but not fat mass (Table 2). When the patients were assigned into two groups based on preoperative thyroid function, thyroidectomy significantly increased body weight and BMI in both groups, whereas a significant increase in skeletal muscle and fat masses was observed only in Group A and Group B, respectively (Table 2), and a significantly higher skeletal muscle mass gain was observed in Group A as compared to Group B (Table 3). These data indicate the preferential effect of thyroid hormone normalization on muscle mass gain. The reason for the significant increase in fat mass in Group B, where the patients were euthyroid due to preoperative medical treatment (Table 2), is unclear at this time, although it may reflect the differences in diet and/or exercise between the two groups that were not evaluated in this study. Furthermore, the significant positive correlation between fT3 and skeletal muscle gain (Figure 1) suggests that the higher the preoperative thyroid hormone level, the greater the effect of total thyroidectomy for Graves’ disease on weight gain. Therefore, these data suggest that the dependence of skeletal muscle mass on thyroid hormone levels is more pronounced than that of fat mass. These data are consistent with other studies that show that thyroid hormones act on nuclear receptors and are involved in skeletal muscle development and growth [14] and that excess thyroid hormones increase protein breakdown, while correction of their levels reduces muscle protein breakdown [15]. A negative association has also been reported between thyroid hormone levels and lower-limb lean body mass in euthyroid individuals [13,16].

As previously mentioned, only a few previous studies have investigated the effect of surgery on body weight and composition in patients with Graves’ disease. Specifically, Lönn et al. reported different temporal effects of surgery on the recovery of muscle and fat masses (an increase in skeletal muscle in the early recovery phase and subcutaneous fat tissue at the later stage) [9], while Dale et al. [11] and Tigas et al. [12] measured only body weight changes preoperatively and postoperatively. 

This study has some limitations despite providing valuable information regarding the body composition of patients with Graves’ disease undergoing total thyroidectomy. First, the sample size was small, and this study did not include time course measurements; therefore, clarifying the difference in recovery time between ATD or radioactive iodine therapy and surgical treatment will be of interest. Moreover, lifestyle changes, dietary intake, and exercise were not assessed, which also play a role in physiological body weight gain and obesity (excess weight gain). Therefore, providing appropriate diet and exercise guidance will be important because some patients with Graves’ disease, particularly those with undesirable body composition (higher body fat and lower lean mass) at presentation, have been reported to gain excess weight, mainly excess fat mass, during treatment [10], which is likely to increase the risk of obesity [17]. In particular, patients with Graves’ disease who underwent total thyroidectomy have been reported to gain more weight than those treated with ATD or radioactive iodine [11]. Proper control of postoperative thyroid function is also essential since patients with Graves’ disease who were euthyroid after subtotal thyroidectomy gained more weight than those who had postoperative hypothyroidism requiring T4 replacement [12]. Therefore, future studies should consider time-course measurements, dietary intake and physical activity, and optimal management of postoperative thyroid function to prevent excessive weight gain and potential obesity, which have been noted in some studies.

## Conclusions

Our study shows that the normalization of thyroid function through total thyroidectomy is associated with weight gain in patients with Graves’ disease, mainly due to an increase in skeletal muscle mass. These data are clinically important as they allow physicians to explain to patients that weight gain after surgical treatment for Graves’ disease is favorable and reassure them of their concerns.
